# Dietary patterns are associated with adolescent growth in China: a latent class analysis

**DOI:** 10.3389/fnut.2025.1690369

**Published:** 2026-01-09

**Authors:** Bingxin Jin, Danyan An

**Affiliations:** 1 Department of Neurology Medicine, Hangzhou Children's Hospital, Hangzhou, China; 2 Department of Respiratory Medicine, Hangzhou Children's Hospital, Hangzhou, China

**Keywords:** dietary patterns, growth, adolescent, Chinese, height

## Abstract

**Background:**

Adolescents’ growth and development are closely related to dietary patterns, yet evidence on the relationship between overall dietary patterns and adolescent growth in contemporary China is limited. This study aimed to identify major dietary patterns among Chinese adolescents and examine their associations with height and height-for-age z-scores (HAZ).

**Methods:**

In this cross-sectional analysis of 2,466 adolescents (age 10–15) from the China Family Panel Studies (2012), dietary patterns derived by latent class analysis were examined in relation to parent-reported height. Dietary assessment used eight binary food-group indicators for past-week consumption; height was reported by a parent or guardian. Associations were estimated using multivariable linear regression and reported as adjusted HAZ differences (and corresponding absolute height differences in centimeters).

**Results:**

Three distinct dietary patterns were identified: Pattern 1 (Meat-Egg Traditional), Pattern 2 (Diverse Protein-Rich), and Pattern 3 (Comprehensive Nutrient-Balanced). In the fully adjusted models, adolescents in Pattern 3 were on average 3.31 cm taller than those in Pattern 1 (*p* < 0.001). Pattern 2 also showed a moderate positive association, with participants being approximately 2.00 cm taller than those in Pattern 1 (*p* = 0.003). Findings for HAZ were consistent with those for absolute height.

**Conclusion:**

These findings suggest that a comprehensive, nutrient-balanced dietary pattern is positively associated with adolescent growth. Promoting nutrient-rich, diversified diets during adolescence could help maximize growth potential and improve developmental outcomes in youth.

## Background

Stunting is a major public health problem in both developed and developing countries (1, 2). According to the World Health Organization (WHO), stunting is defined as a gender-specific height-for-age value of less than two standard deviations below the WHO Child Growth Standards (3). Height-for-age z-scores (HAZ) are widely used to assess this indicator, reflecting cumulative linear growth relative to age and sex. Stunting has long-term impacts on health, cognitive development, educational attainment, and future economic productivity. Although substantial progress has been made in reducing undernutrition globally, an estimated 22% of children under five remain stunted, indicating persistent chronic malnutrition and growth impairment (4). In China, adolescents’ dietary patterns have shifted markedly in recent decades. Traditionally, diets for most Chinese adolescents were dominated by cereals and plant-based foods, but they have gradually incorporated more “Western” elements such as animal-based foods, desserts, fast foods, and sugary snacks. On one hand, greater availability of animal protein and calories has improved growth in height for some, potentially contributing to secular increases in average height. On the other hand, the influx of energy-dense, nutrient-poor foods has driven up rates of overweight and obesity among Chinese youth (5, 6). Understanding how overall dietary patterns relate to linear growth is therefore vital for guiding public health interventions.

Diet plays a fundamental role in linear growth. Beyond adequate caloric intake, the quality of the diet—including diversity and sufficient intake of high-quality protein and micronutrients—is essential for supporting optimal height gain (6, 7). Traditionally, nutrition research has examined single nutrients or foods; however, dietary pattern (DP) analysis has emerged as a complementary approach that captures the combined and potentially synergistic effects of multiple foods as they are consumed in real life (7, 8). Statistical methods such as principal component analysis, factor analysis, cluster analysis, latent class analysis (LCA), and reduced rank regression have been used to identify DPs among adolescents (9–11). This holistic approach can better reflect habitual diet quality and its association with health outcomes, including growth.

Prior studies in China have reported several common dietary patterns among youth, but most have focused on their associations with obesity rather than linear growth. In a nationwide study of Chinese 7–17 year-olds, Zhang et al. found three major patterns: a “Modern” pattern high in milk, fast food, and eggs; a “Traditional North” pattern high in wheat products, tubers, and other grains; and a “Traditional South” pattern high in rice, vegetables, and pork (12). Importantly, the “Modern” and “Traditional North” patterns were each associated with a significantly greater obesity risk compared to the more plant-based “Traditional South” pattern (12). Another study in Beijing preschoolers identified patterns with Sugar-Sweetened Beverages & Snack linked to a 61% higher odds of overweight relative to the traditional pattern (13). However, the implications of these dietary patterns for linear growth (height) are less studied. Most existing literature has focused on weight-related outcomes (e.g., BMI, obesity) (13) or other health and cognitive outcomes (14), rather than height attainment. Height in adolescence is a key indicator of long-term nutritional status – low height-for-age reflects chronic undernutrition or stunting, whereas achieving greater height can indicate better nutrition and health over childhood years (3). It remains unclear whether healthier, diversified diets in adolescence translate into appreciably taller stature by late adolescence, after accounting for genetic and socioeconomic factors, leaving a gap in knowledge about the role of overall dietary patterns in supporting optimal growth.

To address this gap, we used data from the nationally representative China Family Panel Studies (CFPS) to identify dietary patterns among Chinese adolescents using LCA based on food group consumption history. We then examined the association between these dietary patterns and linear growth, as measured by HAZ and absolute height.

## Methods

### Study population and data source

In this cross-sectional study, dietary information, height data, and sociodemographic characteristics of 2,466 adolescents aged 10–15 years were obtained from the 2012 wave of the CFPS. The CFPS, conducted by Peking University, is a nearly nationwide, comprehensive, longitudinal social survey designed to support research on a wide range of social phenomena in contemporary China (15). Participants were recruited from 25 provinces, municipalities, and autonomous regions, covering diverse communities, families, adults, and children, thereby reflecting changes in society, economy, population, education, and health (16).

In the present analysis, we selected adolescents aged 10–15 years as the target population. Of the 3,056 participants initially surveyed in the 2012 CFPS, 236 with missing height data and 354 with missing dietary information were excluded, resulting in a final sample size of 2,466 (Supplementary Figure S1). To assess potential selection bias, we compared the included and excluded participants on major demographic and health characteristics, including age, sex, hukou status, household income, and self-rated health and depression. No significant differences were observed between the two groups, suggesting that the analytic sample was broadly representative of the full baseline population (Supplementary Table S1).

### Dietary assessment and latent class analysis

The dietary patterns were assessed using following question: whther you had eaten these eight groups of foods in the past week, including meat, fish and other aquatic products, fresh vegetables and fruits, dairy products, soy products, eggs, pickled food, and puffed and fried food. Eight dichotomus variables indicating whether participants had eaten the specific kind of food were then generated. Using these eight binary indicators, we conducted LCA to identify underlying dietary pattern classes among adolescents. LCA is a statistical modeling approach that assigns individuals to latent classes such that those in the same class have similar response patterns across the observed dietary indicators (17, 18). We fitted LCA models with 2 through 4 classes and compared model fit statistics (log-likelihood, Akaike Information Criterion (AIC), Bayesian Information Criterion (BIC), sample-size adjusted BIC (ssaBIC), entropy, average posterior probabilities, and class sizes) to determine the optimal number of distinct dietary patterns. If the model was failed to converge, multiple re-estimation attempts using different random seeds (*n* = 10) and alternative optimization algorithms were used to check whether if could converge. The log-likelihood increased with the number of classes, whereas the AIC and BIC reached their lowest values for the three-class model, suggesting better model fit. The average posterior probabilities for each class (ranging from 0.74 to 0.86) demonstrated high within-class homogeneity. Therefore, the 3-class solution provided the best balance of model fit and interpretability, and thus three latent dietary patterns were retained. Each adolescent was assigned a probability of membership in each pattern; they were classified to the pattern of highest probability for descriptive purposes. More details about the model characteristics could be found in Supplementary Table S2. Coding syntax and log document could also be found in the Supplementary Materials.

### Outcomes

In the CFPS, an adolescent’s height was answered by their parents or guardians, and previous studies have demonstrated the effectiveness of self-reported height and weight (19). Height measurements were converted to HAZ by using either the 2007 WHO reference curves (5–19 y) (20). Both crude height and HAZ were used as the outcomes in the following analyses.

### Covariates

Covariates were selected based on their known associations with adolescent growth or dietary habits. These included: age (years, continuous), sex (male or female), and household registration status (urban vs. rural, as a proxy for urbanicity and access to resources) (21, 22). Socioeconomic position was indexed by household per-capita income. We also accounted for general health and psychosocial factors that might confound the diet–height relationship: self-reported health status, the presence of depressive symptoms (assessed via a standard Center for Epidemiologic Studies Depression Scale, CES-D), and physical activity level. Physical activity was assessed by exercise frequency (everyday, two-three times/week, two-three times/month, once a month, never) as reported by the adolescent. Finally, because body composition can influence linear growth and pubertal development, we included weight in the most stringent model to adjust for the adolescents’ overall adiposity or body mass.

### Statistical analysis

There were three major parts of the statistical analysis: (i) descriptive statistics for the socio-demographic characteristics, general health, mental health, physical activity and dietary intake; (ii) identification of dietary patterns; and (iii) assessment of their relationship with adolescents’ growth outcomes.

Observed dietary patterns were described in terms of food consumption histories for all eight food groups investigated in CFPS. These are referred to as ‘empirical dietary patterns’ as opposed to those derived statistically via LCA. LCA was applied directly to the eight food group variables to derive mutually exclusive dietary patterns. No prior variable selection was conducted, as the investigation was designed to capture the major dietary components in Chinese adolescents.

Characteristics of participants were presented as mean and standard deviation (SD) or frequency and percentage. Relationships between absolute height or HAZ and covariates were initalliy examined using independent-sample t tests for binary variables, one-way ANOVA for categorical variables with more than two categories, and Pearson correlation analysis for continuous variables. The association between dietary patterns and growth outcomes—measured by HAZ and absolute height—was examined using multivariable linear regression. Three models were fitted: Model 1 (crude), Model 2 (adjusted for age and sex), and Model 3 (further adjusted for hukou status, household per capita income, self-reported health, depression, and physical activity). The moderating effect of body weight on the association between dietary patterns and height was examined by including an interaction term between dietary pattern and body weight in the regression models after adjusting for other potential confounders. We first fitted ordinary least squares models and tested for heteroskedasticity using the Breusch–Pagan test. Evidence of heteroskedasticity (*p* < 0.05) prompted us to re-estimate the models using heteroskedasticity-robust standard errors. Variance inflation factors (VIFs) were calculated to assess multicollinearity, with all VIFs below 10, indicating no severe multicollinearity (Supplementary Table S3). Model diagnostics were conducted to evaluate the validity of the regression assumptions. Specifically, residual-versus-fitted plots were used to assess linearity and homoscedasticity, and normal Q–Q plots were used to assess the normality of residuals. No substantial deviations from model assumptions were detected. More details could be found in Supplementary materials (Supplementary Figures S2–S5).

All analyses were performed using Stata, version 18.0 (StataCorp LLC, College Station, Texas, USA). A two-sided *p* value <0.05 was considered statistically significant.

## Results

The analytic sample comprised 2,466 adolescents aged 10–15 years (mean age: 12.6 ± 1.7 years), of whom 48.3% were female.

Latent class analysis identified three distinct dietary patterns based on consumption probabilities of eight food groups (Table 1). Pattern 1 (Meat-Egg Traditional) was characterized by predominant meat (probability (P) = 46.5%) and egg consumption (*p* = 55.3%), with limited intake of fish/aquatic products (*p* = 6.8%) and dairy (*p* = 22.1%). Vegetables/fruits remained high (*p* = 90.9%). Pattern 2 (Diverse Protein-Rich) featured near-universal meat (*p* = 97.7%) and vegetable/fruit intake (*p* = 96.9%), with balanced probabilities for eggs (*p* = 77.1%), fish (*p* = 50.7%), dairy (*p* = 42.1%), and soy (*p* = 47.2%). Pattern 3 (Comprehensive Nutrient-Balanced) demonstrated universally high consumption of all core food groups: vegetables/fruits (*p* = 99.6%), soy (*p* = 91.6%), eggs (*p* = 97.0%), dairy (*p* = 81.6%), fish (*p* = 73.8%), and meat (*p* = 99.1%). Processed foods co-occurred at higher probabilities (pickled: *p* = 68.8%, puff/fried: *p* = 78.5%) but did not displace nutrient-dense foods.

**Table 1:** Discriminant dietary patterns identified by latent class analysis.

Patterns	Probability		
	Pattern 1	Pattern 2	Pattern 3
Meat	46.5%	97.7%	99.1%
Fish and other aquatic products	6.8%	50.7%	73.8%
Fresh vegetables and fruits	90.9%	96.9%	99.6%
Dairy products	22.1%	42.1%	81.6%
Soy products	25.6%	47.2%	91.6%
Eggs	55.3%	77.1%	97.0%
Pickled foods	23.7%	23.0%	68.8%
Puff/fried foods	26.0%	34.2%	78.5%

Table 2 summarizes the characteristics of the participants. Height and HAZ were also significantly associated with several covariates. Males had slightly greater mean height (149.2 ± 17.4 cm) and HAZ (−0.6 ± 1.9) than females (147.8 ± 15.0 cm; −0.8 ± 1.9) (*p* < 0.05 for both). Urban adolescents were taller (154.9 ± 13.3 cm) with higher HAZ (0.2 ± 1.5) compared with rural adolescents (146.7 ± 16.6 cm; −0.9 ± 1.9) (*p* < 0.001). Higher household per capita income was positively correlated with both height and HAZ (*p* < 0.001). Self-reported health status showed a graded association with growth indicators: those reporting “very healthy” or “healthy” had higher mean height and HAZ than those rating their health as “average” or “unhealthy” (*p* < 0.01). Adolescents without depressive symptoms were significantly taller and had higher HAZ than those with depression (*p* < 0.001). Physical activity was positively related to growth: participants exercising daily were taller (149.8 ± 15.6 cm) with higher HAZ (−0.6 ± 1.8) than those reporting no activity (144.3 ± 18.4 cm; −1.1 ± 2.1) (*p* < 0.01).

**Table 2:** Characteristics of the adolescents (*n* = 2,466).

Characteristics	N (%) / Mean (SD)	Height		HAZ	
		Height: Mean (SD)	F/ r/t	HAZ Mean (SD)	F/ r/t
Dietary pattern			33.87***		33.31***
Pattern 1	468 (19.0%)	144.4 (17.7)		−1.2 (2.2)	
Pattern 2	1,280 (51.9%)	148.0 (16.1)		−0.7 (1.8)	
Pattern 3	718 (29.1%)	152.1 (15.0)		−0.3 (1.7)	
Age	12.6 (1.7)	148.5 (16.3)	0.59***	−0.7 (1.9)	0.05*
Sex			−2.19*		−2.38*
Female	1,191 (48.3%)	147.8 (15.0)		−0.8 (1.9)	
Male	1,275 (51.7%)	149.2 (17.4)		−0.6 (1.9)	
Household			−10.48***		−13.23***
Rural	1924 (78.0%)	146.7 (16.6)		−0.9 (1.9)	
Urban	542 (22.0%)	154.9 (13.3)		0.2 (1.5)	
Household capita income	8687.1 (13552.3)	148.5 (16.3)	0.11***	−0.7 (1.9)	0.15***
Self-reported health			4.77***		4.38**
Very healthy	681 (27.6%)	148.0 (16.6)		−0.6 (2.0)	
Healthy	943 (38.2%)	149.3 (16.6)		−0.6 (1.9)	
Fairly healthy	652 (26.4%)	149.1 (15.5)		−0.6 (1.7)	
Average	163 (6.6%)	144.4 (14.4)		−1.2 (1.7)	
Unhealthy	27 (1.1%)	141.7 (19.6)		−1.2 (2.5)	
Depression			6.04***		7.40***
No	2,158 (87.5%)	149.3 (15.7)		−0.6 (1.8)	
Yes	308 (12.5%)	143.3 (19.5)		−1.4 (2.2)	
Physical activity			6.07***		6.03**
Everyday	769 (31.2%)	149.8 (15.6)		−0.6 (1.8)	
Two-three times/week	1,154 (46.8%)	148.9 (16.1)		−0.6 (1.9)	
Two-three times/month	206 (8.4%)	147.7 (16.2)		−0.8 (1.8)	
Once a month	71 (2.9%)	147.3 (16.2)		−0.8 (1.9)	
Never	266 (10.8%)	144.3 (18.4)		−1.1 (2.1)	
Weight	78.6 (24.3)	148.5 (16.3)	0.61***	−0.7 (1.9)	0.43***

*P < 0.05; **P < 0.01; ***p < 0.001. SD, standard deviation; HAZ, height-for-age z-scores.

Tables 3, 4 presents the results of multivariable regression analyses examining the association between dietary patterns and HAZ/absolute height. In the fully adjusted model (Model 3), which additionally controlled for household type (urban/rural), household per capita income, self-reported health, depression, physical activity, and weight, both Pattern 2 and Pattern 3 remained significantly associated with higher HAZ. Specifically, Pattern 2 was associated with a 0.26 higher HAZ (95% CI: 0.09, 0.44; *p* = 0.003), while Pattern 3 was associated with a 0.39 higher HAZ (95% CI: 0.19, 0.59; *p* < 0.001) compared to Pattern 1. Similar results were observed when using height (cm) as the outcome, indicating that the positive associations between healthier dietary patterns and growth are consistent across both relative (z-score) and absolute height measures. These findings were further explored by examining potential interactions with body weight. Moreover, interaction analyses between dietary patterns and body weight on HAZ and height are presented in Supplementary Table S4. The interaction terms were negative and statistically significant, suggesting that the positive associations between healthier dietary patterns and growth were attenuated as body weight increased.

**Table 3:** The impact of dietary patterns on height-for-age z-scores in multivariate regression.

Models	Coefficient	*P*	95% CI	
			Lower 95% CI	Upper 95% CI
Model 1				
Dietary pattern (ref: Pattern 1)				
Pattern 2	0.48	*P* < 0.001	0.28	0.68
Pattern 3	0.90	*P* < 0.001	0.68	1.11
Model 2				
Dietary pattern (ref: Pattern 1)				
Pattern 2	0.47	*P* < 0.001	0.27	0.67
Pattern 3	0.88	*P* < 0.001	0.66	1.10
Model 3				
Dietary pattern (ref: Pattern 1)				
Pattern 2	0.28	0.004	0.09	0.44
Pattern 3	0.45	*P* < 0.001	0.19	0.59

Model 1, crude model; Model 2, adjusted for age and sex; Model 3: adjusted for age, sex, household, household capita income, self-reported health, depression, and physical activity.

**Table 4:** The impact of dietary patterns on absolute height in multivariate regression.

Models	Coefficient	*P*	95% CI	
			Lower 95% CI	Upper 95% CI
Model 1				
Dietary pattern (ref: Pattern 1)				
Pattern 2	3.59	*P* < 0.001	1.88	5.29
Pattern 3	7.70	*P* < 0.001	5.83	9.58
Model 2				
Dietary pattern (ref: Pattern 1)				
Pattern 2	3.30	*P* < 0.001	1.93	4.67
Pattern 3	6.26	*P* < 0.001	4.75	7.77
Model 3				
Dietary pattern (ref: Pattern 1)				
Pattern 2	2.00	0.003	0.66	3.06
Pattern 3	3.31	*P* < 0.001	1.48	4.21

Model 1, crude model; Model 2, adjusted for age and sex; Model 3, adjusted for age, sex, household, household capita income, self-reported health, depression, and physical activity.

## Discussion

In this nationally representative sample of Chinese adolescents aged 10–15 years, we identified three distinct dietary patterns using LCA. Our findings indicate that greater dietary diversity was positively associated with linear growth. Adolescents classified in the Comprehensive Nutrient-Balanced pattern—characterized by near-universal intake of vegetables/fruits, dairy, soy, eggs, meat, and fish—had significantly higher HAZ than those in the Meat–Egg Traditional pattern.

Our results align with a growing body of evidence linking diet quality and diversity to improved growth in children and adolescents. For example, a large study of Israeli adolescents found that a “junk food” dietary pattern (high in sweets and processed snacks) was associated with lower height-for-age, whereas healthier patterns correlated with normal growth (23). Similarly, research in other populations has shown that children with higher diet quality scores or greater dietary diversity tend to be taller, whereas those consuming more sugar-rich and less nutrient-dense foods are at risk of poorer growth (24). In the United States, children with low HAZ were found to consume more soft drinks and sugary snacks, while those with higher HAZ had diets richer in milk, fruits, and other nutrient-dense items (24). These findings underscore that adequate nutritional intake and a diverse, nutrient-rich diet are crucial for optimal height gain (25). Notably, Pattern 3 included a high probability of dairy consumption – a food group well known to promote linear growth due to its calcium and high-quality protein content. Higher intakes of milk and dairy products has been linked to reduced stunting and improved height in children (26, 27). Eggs, another key protein-rich food, were also consumed much more frequently in Pattern 3 than in the traditional pattern. Regular egg intake has been associated with better linear growth in childhood; for instance, a randomized trial reported that children given eggs daily grew taller than those with infrequent egg consumption (28, 29). Thus, the positive height outcomes observed for Patterns 2 and 3 are biologically plausible, as these diets supplied more protein (meat, eggs, dairy, soy) and micronutrients essential for bone growth (calcium, vitamins) than the limited Meat-Egg Traditional pattern. It is worth noting that Pattern 3 also had higher probabilities of processed foods (pickled and fried items), yet those did not offset the benefits of its nutrient-dense components. This suggests that overall diet quality – particularly the presence of diverse wholesome foods – can outweigh the occasional inclusion of less healthy items when it comes to linear growth. However, the long-term health implications of the processed foods in Pattern 3 (beyond height outcomes) remain an important consideration.

In this study we applied LCA to derive dietary patterns, which is a relatively novel approach in nutritional epidemiology. Although LCA is a well-established method for pattern recognition, its use in identifying dietary patterns has been surprisingly scarce in prior research (11, 30). LCA offers several advantages over traditional techniques (such as principal component or factor analysis) for dietary pattern analysis. First, it can identify latent subgroups of individuals with similar eating behaviors, accounting for unobserved heterogeneity in food intake preferences across the population. Second, LCA does not assume multivariate normality of input variables, an assumption that is often violated with skewed dietary intake data. Third, LCA provides intuitive outputs – each latent class comes with estimated probabilities of consuming each food group – which facilitates interpretation and the calculation of epidemiologic effect estimates for belonging to a given dietary class. Cluster analysis can identify groups of individuals with similar diets, but it is typically applied to continuous consumption variables and often requires subjective decisions on the number of clusters. In contrast, LCA objectively tests model fit to determine the optimal number of classes and is naturally well-suited for the binary/categorical frequency data from food frequency questionnaires. Given these advantages, LCA proved to be an appropriate and powerful tool in our study for uncovering meaningful dietary patterns that might have been overlooked by other methods.

To our knowledge, this study is the first to characterize adolescent dietary patterns in China using LCA and to demonstrate their relationship with growth outcomes. Previous studies in Chinese adolescents have typically used methods like factor analysis or diet quality indices to examine nutrition and health, but our LCA approach reveals distinct diet profiles existing within the population. The identification of a “Comprehensive Nutrient-Balanced” pattern associated with superior growth adds new evidence that diet diversification and quality are key determinants of adolescent height even in the context of contemporary China’s nutrition transition. Our findings support the emphasis of current food-based dietary guidelines on dietary diversity and suggest that nutrition interventions aiming to improve child and adolescent growth should incorporate education and strategies to increase the intake of under-consumed nutritious foods.

### Limitations

This study is cross-sectional and cannot establish temporality or causality; the observed relationships should therefore be interpreted as associations. Height was parent-reported rather than directly measured, which may introduce measurement error. Dietary exposure was assessed using eight binary past-week indicators without portion sizes or total energy estimates, limiting ability to quantify intake. Important potential confounders—most notably parental (genetic) height, pubertal stage, and total energy intake—were not available for adjustment; these omissions could bias the estimated associations. Finally, a substantial number of participants were excluded due to missing data; a flow diagram and sensitivity analyses (Supplementary Table S1) are provided to assess potential selection bias.

## Conclusion

In summary, this study identified three dietary patterns among Chinese adolescents using LCA, and demonstrated that adolescents consuming a comprehensive, balanced diet had significantly better growth (height) than those consuming a more monotonous diet. A pattern characterized by high intakes of diverse, nutrient-dense foods (including vegetables, fruits, dairy, eggs, and lean proteins) was associated with taller stature and improved height, even after accounting for socioeconomic and lifestyle factors. These findings underscore the critical role of diet quality in supporting adolescent growth. Improving the diet diversity and nutritional quality of meals for adolescents should be a priority. Food-based dietary guidelines and nutrition interventions that encourage the intake of a variety of healthful foods (while limiting less nutritious options) could be integral to strategies for preventing growth faltering and stunting in children. By fostering healthier eating patterns early in life, we can help ensure that adolescents not only attain their optimal height potential but also lay a foundation for better overall health.

## Data Availability

Publicly available datasets were analyzed in this study. The original data in this study was obtained from the Institute of Social Science Survey of Peking University and are available at: https://www.isss.pku.edu.cn/cfps/index.htm.
