# Supplementary material for: Dietary patterns are associated with adolescent growth in China: a latent class analysis
Source: Front Nutr. 2026 Jan 9;12:1690369. doi: 10.3389/fnut.2025.1690369 (PMC12827119; doi:10.3389/fnut.2025.1690369)
Supplement: Supplementary file 1 [file Supplementary_file_1.docx]

**Supplementary Materials**

**1. Supplementary Tables**

**Table S1.** Comparison of included and excluded participants on key characteristics

|  | Included sample | Excluded sample |
| --- | --- | --- |
| Age (Mean, SD) | 12.6 (1.7) | 12.4 (1.8) |
| Sex |  |  |
| Female | 1,191 (48.3%) | 108 (45.0%) |
| Male | 1275 (51.7%) | 132 （55.0%） |
| Hukou |  |  |
| Rural | 1924 (78.0%) | 536 (90.9%) |
| Urban | 542 (22.0%) | 54 (9.2%) |
| Household capita income (log transferred) | 8.5 (1.3) | 8.2 (1.3) |
| Self-reported health |  |  |
| Very healthy | 681 (27.6%) | 60 (27.4%) |
| Healthy | 943 (38.2%) | 80 (36,5%) |
| Fairly healthy | 652 (26.4%) | 60 (27.4%) |
| Average | 163 (6.6%) | 13 (5.9%) |
| Unhealthy | 27 (1.1%) | 6 (2.7%) |
| Depression |  |  |
| No | 2158 (87.5%) | 466 (78.9%) |
| Yes | 308 (12.5%) | 124 (21.0%) |

**Table S2.** Model fit indices for latent class models with two and three classes

| Indicators | Two classes model | Three classes model |  |
| --- | --- | --- | --- |
| Log-likelihood | -10483.96 | -10413.48 |  |
| AIC | 21001.92 | 20878.97 |  |
| BIC | 21100.69 | 21030.04 |  |
| Entropy | 0.61 | 0.55 |  |
| Average posterior probabilities | Class 1: 0.89  Class 2: 0.11 | Class 1: 0.86 Class 2: 0.74 Class 3: 0.83 |  |
|  |  |  |  |
|  |  |  |  |
| Class sizes (%) | Class 1: 53.8% Class 2: 46.2% | Class 1: 24.1% Class 2: 45.6% Class 3: 30.2% |  |
|  |  |  |  |
|  |  |  |  |

# AIC, Akaike Information Criterion; BIC, Bayesian Information Criterion.

**Table S3**: VIF of the regression models

|  | Height | | HAZ | |
| --- | --- | --- | --- | --- |
| Variable | VIF | 1/VIF | VIF | 1/VIF |
| Dietary pattern (ref: Pattern 1) |  |  |  |  |
| Pattern 2 | 1.85 | 0.54 | 1.85 | 0.54 |
| Pattern 3 | 1.97 | 0.51 | 1.97 | 0.51 |
| Age | 1.02 | 0.98 | 1.02 | 0.98 |
| Sex | 1.01 | 0.99 | 1.01 | 0.99 |
| Hukou | 1.12 | 0.89 | 1.12 | 0.89 |
| Household capita income | 1.11 | 0.90 | 1.11 | 0.90 |
| Self-reported health (ref:Very healthy) |  |  |  |  |
| Healthy | 1.49 | 0.67 | 1.49 | 0.67 |
| Fairly healthy | 1.46 | 0.68 | 1.46 | 0.68 |
| Average | 1.18 | 0.85 | 1.18 | 0.85 |
| Unhealthy | 1.03 | 0.97 | 1.03 | 0.97 |
| Depression | 1.03 | 0.97 | 1.03 | 0.97 |
| Physical activity (ref: Everyday) | |  |  |  |
| Two-three times/week | 1.35 | 0.74 | 1.35 | 0.74 |
| Two-three times/month | 1.17 | 0.86 | 1.17 | 0.86 |
| Once a month | 1.07 | 0.93 | 1.07 | 0.93 |
| Never | 1.24 | 0.81 | 1.24 | 0.81 |
| Mean VIF | 1.27 |  | 1.27 |  |

**Table S4**. Results of interaction analyses between dietary patterns and body weight in relation to height-for-age z-scores and absolute height

|  | Coefficient | P | 95% CI | |
| --- | --- | --- | --- | --- |
|  |  |  | Lower 95% CI | Upper 95% CI |
| Height-for-age z-scores: |  |  |  |  |
| Dietary pattern (ref: Pattern 1)#Weight |  |  |  |  |
| Pattern 2 # Weight | -0.02 | P<0.001 | -0.02 | -0.01 |
| Pattern 3 # Weight | -0.03 | P<0.001 | -0.03 | -0.02 |
| Height: |  |  |  |  |
| Dietary pattern (ref: Pattern 1)#Weight |  |  |  |  |
| Pattern 2 # Weight | -0.11 | P<0.001 | -0.16 | -0.06 |
| Pattern 3 # Weight | -0.17 | P<0.001 | -0.22 | -0.11 |

**2. Supplementary Figures**

**Figure S1.** Flowchart of participant selection in the study.

**Figure S2.** Residuals and fitted values for height regression model


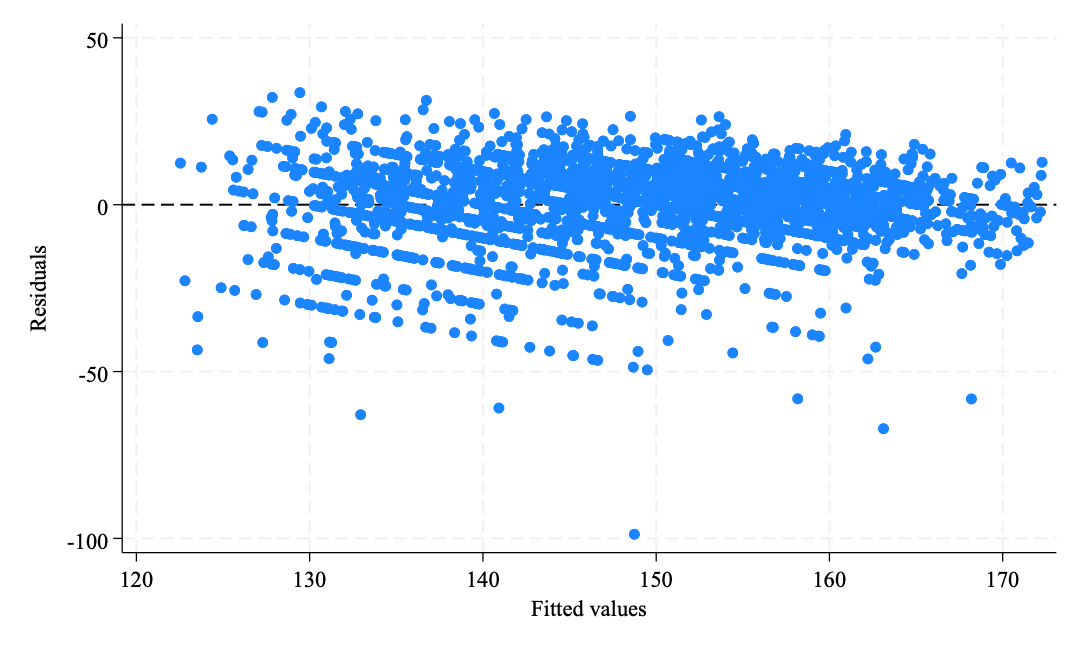


**Figure S3.** Q-Q plots for height regression model


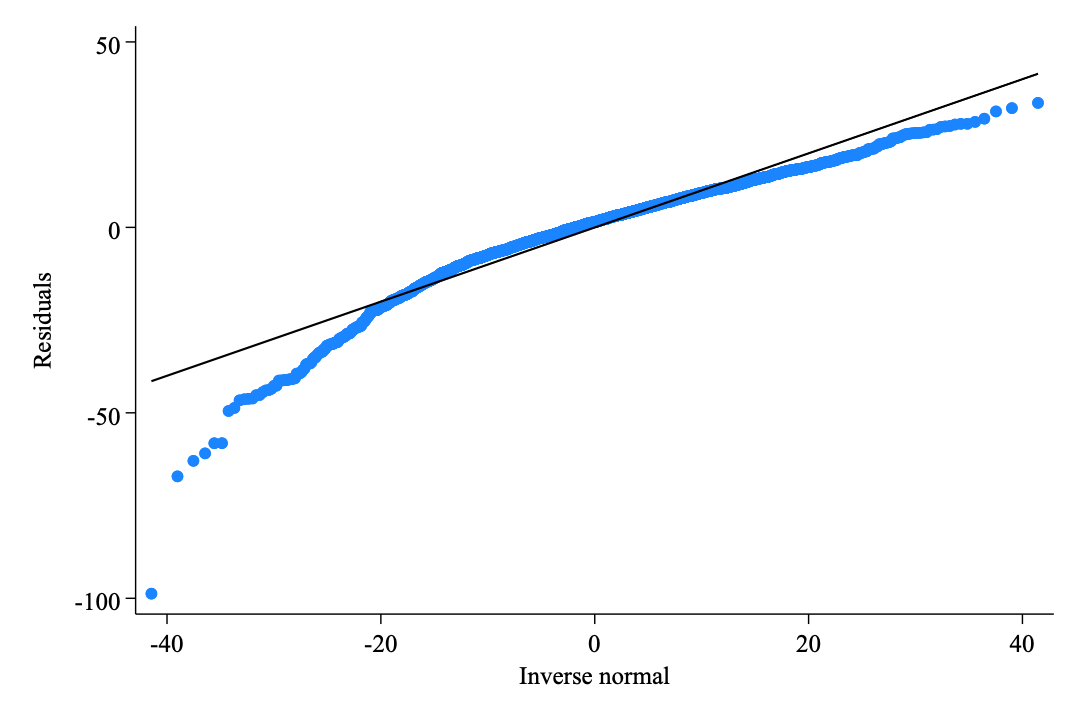


**Figure S4.** Residuals and fitted values for HAZ regression model


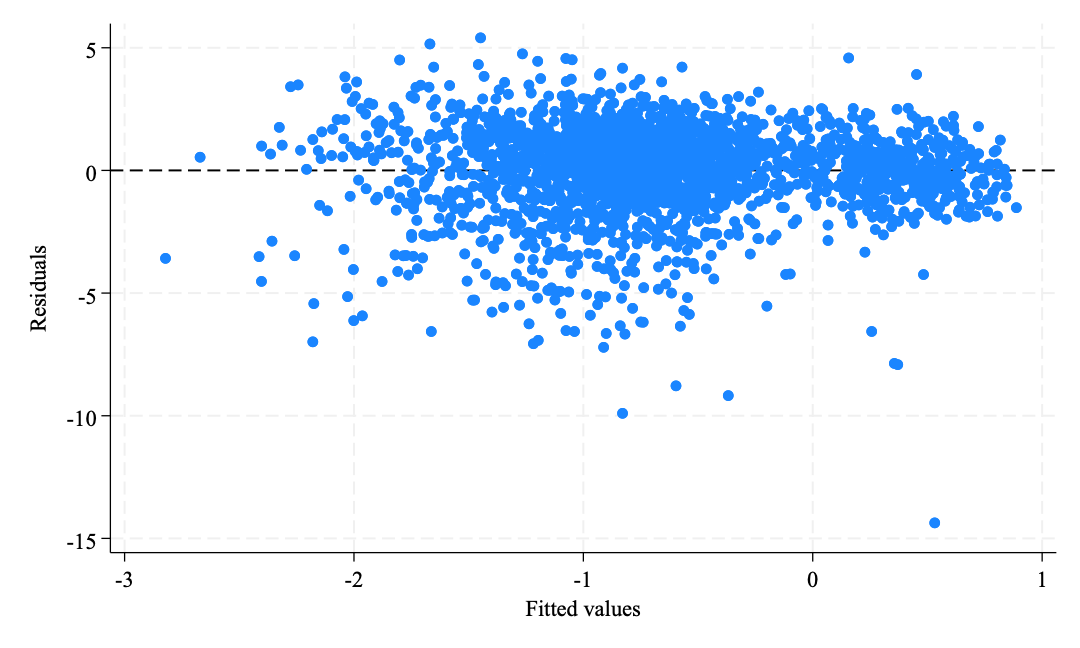


**Figure S5.** Q-Q plots for HAZ regression model

**
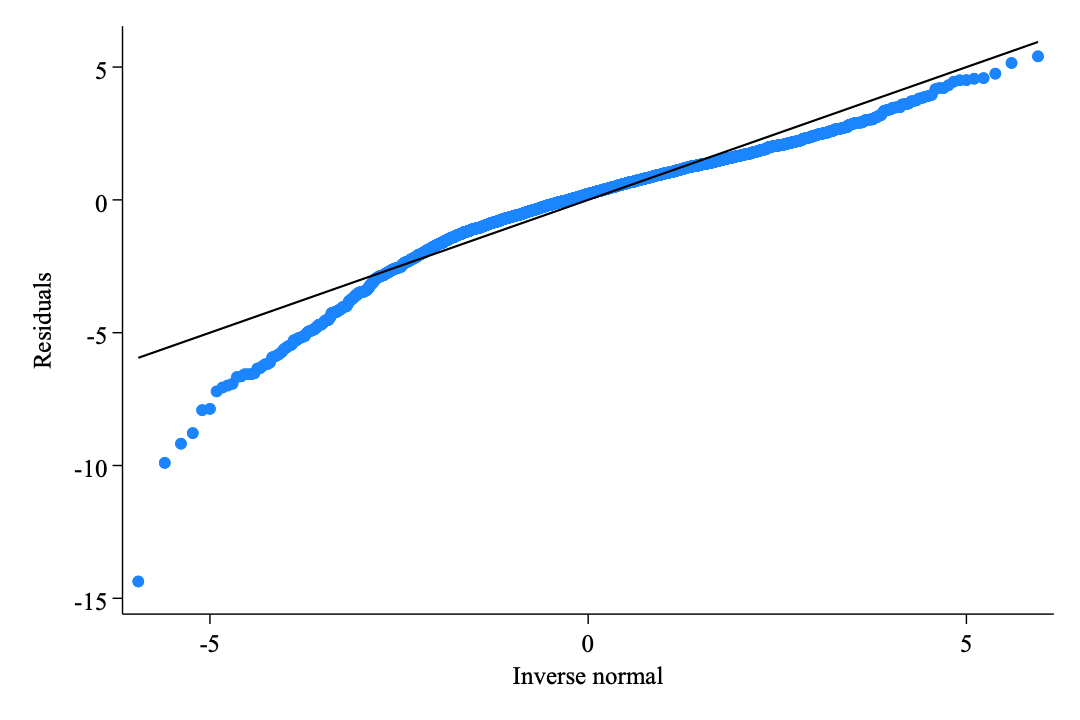
**

**3. LCA syntax**

**4. LCA Log files**
